# Impact of Fixed Orthodontic Appliance and Clear Aligners on the Periodontal Health: A Prospective Clinical Study

**DOI:** 10.3390/dj8010004

**Published:** 2020-01-02

**Authors:** Ada Carolina Pango Madariaga, Rosaria Bucci, Roberto Rongo, Vittorio Simeon, Vincenzo D’Antò, Rosa Valletta

**Affiliations:** 1 Department of Neuroscience, Reproductive Science and Oral Science, Division of Orthodontics, University of Naples Federico II, 80131 Naples, Italy; apangom@gmail.com (A.C.P.M.); roberto.rongo@unina.it (R.R.); vincenzodanto@gmail.com (V.D.); valletta@unina.it (R.V.); 2 Department of Mental Health and Preventive Medicine, Medical Statistics Unit, University of Campania “Luigi Vanvitelli”, Largo Madonna Delle Grazie, 80138 Naples, Italy

**Keywords:** fixed orthodontic appliances, clear aligners, oral hygiene, dental plaque, gingival health

## Abstract

This study aimed to evaluate the periodontal health of orthodontic patients with supportive periodontal therapy in a 3 month follow-up. The sample comprised 20 patients (mean age 20.6 ± 8.1 years) in treatment with multibracket fixed appliances (fixed group—FG) and 20 patients (mean age 34.7 ± 12.5 years) in treatment with clear aligners (clear aligners group—CAG). At baseline (T0) and after 3 months (T1), probing depth (PD), plaque index (PI), bleeding on probing (BOP), and gingival recession (REC) were measured. Patients were trained to perform an individualized tooth brushing technique, and every 2 weeks they were re-called to reinforce the oral hygiene instructions. The intra-group comparisons (T1 vs. T0) were calculated with the Wilcoxon signed-rank test, while a linear regression model was used for the inter-group comparisons (FG vs. CAG). The significance level was set at *p* < 0.05. Statistically significant decrease in both groups was found for PD (FG: Δ, −9.2 inter-quartile range (IQR), −22.5, −5.5; CAG: Δ, −12.6 IQR, −25.4, −4.8), BOP (FG: Δ, −53.5 IQR, −70.5, −37; CAG: Δ, −37.5 IQR, −54.5, −23), and PI (FG: Δ, −17.5 IQR, −62.5, 14.5; CAG: Δ, −24 IQR, −49.5, −5). The result of the linear regression models suggested that the type of appliance did not have any effects on the improvement of periodontal variables. Therefore, patients undergoing orthodontic treatment with fixed appliances and clear aligners did not show differences in gingival health when followed by a dental hygienist.

## 1. Introduction

The main etiological factor in the development of gingivitis is the supragingival dental plaque along the gingival margin. Gingivitis is the inflammatory response of the gingival tissues to the metabolic products and pathogenic toxins of bacteria found in the oral biofilm. The inflammatory change of supragingival plaque is a strong predisposing factor for disease progression. Although gingivitis does not always progress to periodontitis, periodontitis is always preceded by gingivitis [1,2].

Periodontal diseases are very common problems in children, adolescents, and adults. Among school children from primary school, almost 55% of individuals experienced some periodontal problems [3]. Also, epidemiological studies revealed a prevalence range of 35%–41% for moderate periodontitis and of 10%–41% for severe periodontitis [4,5]. Furthermore, it has been reported that the prevalence of aggressive and advanced forms of periodontitis is 10%–14% and it increases in the age groups from 35–44 years. [6,7]. Accordingly, more than 70% of adults presented some form of periodontal disease. Therefore, periodontal treatment is a crucial step, before starting any orthodontic treatment, to restore and maintain the health of the supporting periodontal tissues [8].

Malocclusion is a frequent finding among adolescents and adult, [9,10], and fixed orthodontic therapy is the most common approach for treating different types of malocclusions. However, despite the effectiveness of the multibracket fixed therapy, this type of treatment makes the dental hygiene procedures more difficult due to the presence of brackets, bands, and archwires [11]. Therefore, it prevents optimal hygiene of the oral cavity and it promotes the accumulation of dental biofilm, which, in turn, can lead to the development of white spot lesions, caries, and can seriously damage the periodontium [8]. In particular, it has been shown that patients wearing fixed orthodontic appliances present the highest accumulation of bacterial plaque in the gingival margin and behind the archwires of the maxillary lateral incisors and canines. The frequency of tooth brushing and the motivation for the orthodontic treatment is significantly associated with a reduction of dental biofilm in subjects undergoing fixed orthodontic therapy [12].

Clear aligner treatment has been introduced in the last decades to satisfy the aesthetic and comfort requirements of adult orthodontic patients. This treatment is based on removable thermoplastic splints covering all the teeth and part of the marginal aspects of the gingiva, which progressively move the teeth into an ideal position [13]. Thanks to the satisfactory mechanical proprieties of these devices and to the valuable progresses of the aligners technology, nowadays this therapy is suitable for the correction of a wide spectrum of malocclusions [14]. Results from a systematic review revealed that periodontal indices, as well as the quantity and quality of plaque, are better during clear aligner treatment than during fixed orthodontic therapy [15]. 

During the orthodontic treatment, the dental hygienist must provide the patient with adequate tools to perform regular and satisfactory home oral hygiene. Since the development of a patient’s oral hygiene skills requires teaching and close guidance during repeated visits, dental hygienists have a primary role in the acquisition of such skills [16]. Furthermore, to achieve continuous patient compliance throughout the treatment, dental hygienists should perform periodic check-ups and reinforce the home hygiene techniques by means of auxiliary dental products [16,17]. The available scientific evidence shows that the intervals of periodontal support therapy should be individualized to the patient’s need. For example, a recall interval every 3 months for all patients after periodontal therapy is weak [18]. Finally, individualized education, and clinical and motivational strategies should be adopted to raise the awareness of the importance of brushing teeth regularly to maintain a healthy condition for teeth and gingiva, which is crucial for orthodontic patients [8,19]. 

The aim of this study was to evaluate the periodontal health of patients undergoing fixed orthodontic and clear aligner therapy with a supportive periodontal therapy after a 3 month follow-up. The null hypothesis was that there was not difference in the periodontal health of patients with fixed orthodontic and clear aligner therapy, even after the intervention of the dental hygienist.

## 2. Materials and Methods

The study sample comprised 40 consecutive patients (age >12 years) with permanent dentition (26 females, 14 males, mean age 27.6 ± 12.6), recruited among patients already undergoing the orthodontic treatment at the Section of Orthodontics and Temporomandibular Disorders of the University of Naples Federico II (Naples, Italy). All the patients were treated by postgraduate students of the School of Orthodontics. Twenty patients (mean age 20.6 ± 8.1 years) presented ongoing multibracket fixed therapy (Fixed Group—FG), whereas 20 patients (mean age 34.7 ± 12.5 years) were in treatment with clear aligners (Clear Aligners Group—CAG). For the FG, metal brackets (Mini Sprint, Forestadent^®^, Pforzheim, Germany) and 0.016″ NiTi archwire (Biostarter^®^, Pforzheim, Germany) were used. For the CAG, aligners were made of polyethylene terephthalate glycol copolyester (PET-G), 0.75 mm thick (AirNivol S.r.l, Pisa, Navacchio, Italy). Exclusion criteria included diseases requiring premedication to perform periodontal probing, systemic diseases that can influence the activity of periodontal disease, individuals taking drugs that affect the periodontal status, patients with removable prostheses, and pregnant or breastfeeding women.

All patients were fully informed about the nature of the study and signed an informed consent. The investigations were carried out following the rules of the Declaration of Helsinki of 1975, revised in 2013, and approval was obtained from the Ethics Committee of the University of Naples Federico II (protocol and acceptance number 119/19) before undertaking the research.

### 2.1. Periodontal Assessment and Clinical Procedure

At baseline (T0), periodontal charting was performed, recording gingival biotype, plaque index (PI), bleeding on probing (BOP), probing depth (PD), and gingival recessions (REC). The gingival biotype was evaluated, based on the transparency of the periodontal probe through the gingival margin of the tooth while probing the mid buccal sulcus of both central, lateral incisors and canine, both maxillary and mandibular. If the outline of the probe could be seen through the gingival margin, it was categorized as “thin”; if not, it was categorized as “thick” [20]. All the variables were recorded by one expert operator (periodontist), using a millimeter periodontal probe (15 mm North Carolina probe), inserted in the gingival sulcus with a force of about 0.25 Newton. Subsequently, one trained dental hygienist performed supra- and subgingival scaling, to remove the dental biofilm and calculus. Finally, all patients were trained to perform an individualized tooth brushing technique. Every two weeks, the patients were re-called to reinforce the home oral hygiene instructions. Motivation and oral hygiene instructions and reinforcement were provided by the same professional dental hygienist.

After 3 months (T1), the periodontal health check-up was repeated using the same indices.

### 2.2. Sample Size

A sample size analysis was performed before recruitment. The primary outcome measure of this study was the PI. Based on a previous investigation [21], it was assumed that a clinical significant difference in PI was 5% and that the two groups share a common standard deviation of 5%. A sample size including 17 subjects per group was sufficient to detect between-group differences in PI (α = 0.05 and 1 − β = 0.8). To avoid underpowered study due to drop out, the sample size was increased to 20 patients for each group. 

### 2.3. Statistical Analysis

Descriptive statistics on age, gender, number of sites, gingival biotype, and baseline characteristics were performed at baseline. Shapiro–Wilk test was performed to evaluate variable distribution. According to the distribution, continuous variables were reported as mean (M) and standard deviation (SD) or median and inter-quartile range (IQR), and the between-groups difference (CAG vs. FG) was computed with unpaired Student’s t-test or Mann–Whitney test. Categorical variables were reported as absolute number and percentages, and the between-groups difference (CAG vs. FG) was computed with chi-square test or Fisher exact test. The comparisons between T1 vs. T0 (the difference T1 − T0 was named delta Δ), in each specific treatment group, for PD, PI, BOP, and REC variables were performed with a paired test for asymmetric distribution (Wilcoxon signed-rank test). To test if the difference Δ of each periodontal variable was influenced by type of treatment or by other variables, a linear regression model was performed. The beta coefficient, with 95% confidence interval (95% CI), of CAG vs. FG was reported. Four different models were proposed: Model 1, CAG vs. FG unadjusted; Model 2, model 1 + adjustment according to the baseline values; Model 3, model 2 + adjustment according to age and number of sites; Model 4, model 2 + adjustment according to propensity score. The propensity scores were estimated by fitting the logistic regression model with different treatment method (CAG or FG) as dependent variable. The level of statistical significance was set at *p* < 0.05. Statistical analysis was performed using STATA version 14.0.

## 3. Results

At the baseline (T0), a statistically significant difference between the two groups was found regarding the age (*p* = 0.0001), with patients belonging to the CAG being older than those belonging to the FG (CAG 34.7 ± 12.5 years; FG 20.6 ± 8.1 years). Moreover, the number of sites examined was statistically different (*p* = 0.03), as shown in Table 1. In addition, 75% of all individuals examined had a thick gingival biotype. Furthermore, BOP was significantly increased in the FG as compared with CAG (FG = Median 77 (IQR 56.5, 85); CAG = Median 55.5 (IQR 39.5, 70); *p* = 0.006), while REC was more present in the CAG (CAG = Median 22.2 (IQR 7.1, 32.9) than in FG (FG = Median 4.4 (IQR 0, 14.7); *p* = 0.016).

**Figure 1:**
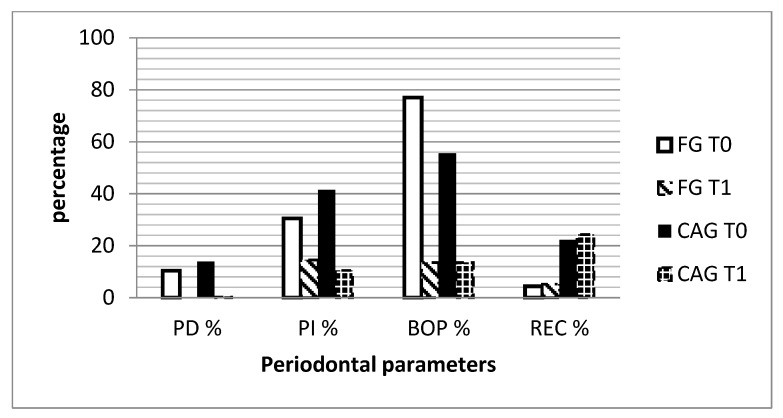
Graph describing the mean % of the four periodontal parameters assessed at baseline (T0) and at 3 month follow-up (T1) in the two groups. FG: fixed group; CAG: clear aligners group; PD: probing depth, PI: plaque index, BOP: bleeding on probing; REC: gingival recession.

The intra-group comparisons (T1 vs. T0) showed statistically significant decreases in both groups for PD (FG: Δ, −9.2 IQR, −22.5, −5.5; *p* = 0.0001; CAG: Δ, −12.6 IQR, −25.4, −4.8; *p* = 0.0002), BOP (FG: Δ, −53.5 IQR, −70.5, −37; *p* = 0.0001; CAG: Δ, −37.5 IQR, −54.5, −23; *p* = 0.0002), and PI (FG: Δ, −17.5 IQR, −62.5, 14.5; *p* = 0.04; CAG: Δ, −24 IQR, −49.5, −5; *p* = 0.002) (Table 2). REC increased significantly only in the FG (Δ, 1.3 IQR, 0, 3.4; *p* = 0.006), as shown in Table 2 and Figure 1.

The four linear regression models of difference Δ confirmed that the type of orthodontic appliances did not have any effects on the improvement of each periodontal variable (Model 2: ΔPD, β 1.2, 95% CI −1.8 to 4.1, *p* = 0.43; ΔPI, β −0.6, 95% CI −12.9 to 11.7, *p* = 0.92; ΔBOP, β −0.45, 95% CI −10.6 to 9.7, *p* = 0.93; ΔREC, β −0.45, 95% CI −6.2 to 5.2, *p* = 0.87). This finding was not affected by differences between groups such as patient’s age and number of sites. (Model 3: ΔPD, β 2.9, 95% CI −0.8 to 6.7, *p* = 0.12; ΔPI, β −3.1, 95% CI −18.5 to 12.3, *p* = 0.68; ΔBOP, β −2.03, 95%CI −11.3 to 15.4, *p* = 0.76; ΔREC, β −4.03, 95% CI −10.2 to 2.1, *p* = 0.19), as shown in Table 3.

## 4. Discussion

The present study aimed to evaluate the periodontal health of patients undergoing fixed orthodontic and clear aligner therapy with a supportive periodontal therapy after a 3 month follow-up. The results confirm the null hypothesis, as no difference was observed in the periodontal health of the two groups of patients when followed by a dental hygienist for 3 months. Indeed, these findings showed that the patients’ periodontal status improved in both groups after the intervention of the professional dental hygienist and no significant effect of the appliance was found.

The oral cavity in colonized by a complex ecosystem of oral microbiota [22]. The problem of the lack of adequate microbial plaque removal takes on greater dimensions when undergoing orthodontic treatment [23,24]. Therefore, the orthodontic patient not only requires greater professional assistance, but also precise and individualized instructions for home oral hygiene, which must be continuous and rigorous, given the presence of orthodontic devices that lead to a potential worsening of conditions of the oral cavity until the onset of diseases. In fact, the present study showed that patients undergoing orthodontic treatment presented gingivitis associated to dental plaque, in accordance to the new classification of periodontal and peri-implant diseases and conditions [25].

In the scientific literature, there is still debate on the influence of clear aligners on oral hygiene. Miethke and co-workers showed that the plaque index of patients treated with clear aligners was significantly lower than that of patients with conventional fixed orthodontics, at the different time points. Nevertheless, the oral hygiene improved in both groups during the entire course of the study [26]. A study by Levrini and co-workers pointed out that patients undergoing orthodontic treatment with clear aligners prompted a lower total biofilm mass accumulation in the short term when compared with patients in treatment with fixed orthodontic appliances, suggesting the use of clear aligners as a first treatment option in patients who are at risk of developing periodontal diseases [27]. Two recent meta-analyses underlined that clear aligners should be used in patients with high risk of gingival inflammation, but the level of evidence was very low and more high-quality studies are required to corroborate these results [28,29]. Interestingly, in the current survey, the patients were enrolled in the study as they were already undergoing orthodontic treatment (either multibracket therapy or clear aligners therapy), and they were naive from any individualized oral hygiene instruction. The comparison at the baseline (T0) showed increased BOP in the fixed orthodontic group, supporting that when no adequate information is provided to the patients, poorer oral hygiene can be observed in patients wearing multibracket appliances. However, the supportive therapy provided by the professional dental hygienist determined a dramatic improvement in the periodontal health of both groups of patients, independently from the kind of appliance. These results suggest that when appropriate oral hygiene instruction and motivation are offered to the patients, the type of orthodontic treatment has no effect on periodontal health. Furthermore, these findings are in accordance with recent prospective randomized control trials by Chhibber and co-authors that pointed out no evidence of differences in oral hygiene levels among clear aligners, self-ligated brackets, and conventional elastomeric ligated brackets after 18 months of active orthodontic treatment [30]. 

Differently from the previous studies, the current survey gave great importance not only to professional oral hygiene, but also to the motivation of the patients’ home hygiene with regular check-ups (every 2 weeks) and by personalizing home-hygiene techniques. This result agrees with previous studies in which the importance of motivation in orthodontic patients was addressed [31,32]. Furthermore, regular check-ups are crucially important in order to perform appropriate differential diagnosis in presence of gingival bleeding [33]. 

Interestingly, in the current study, changes of REC were observed only in the FG groups. One possible explanation was the higher degree of dental expansion due to the fixed orthodontic treatment related to the standard arch-form of the wire [19]. 

The current study presents several strengths. First, periodontal assessments, professional hygiene and motivation, training, and check-ups were given to the patients by only two trained clinicians. This avoided bias due to differences in operator performance. Second, since the first step toward improving oral hygiene is patient compliance, monitoring gingival health and reinforcing the patient’s individualized tooth brushing techniques were performed every 2 weeks to increase patient awareness of the importance of good oral hygiene. The study also has some limitations. First, the baseline age of patients was statistically significant different between the two groups due to the increasing number of adult patients asking for aesthetic orthodontic therapy. Moreover, no data on the smoking status were collected at the baseline. Finally, the reported data have been collected with a short follow-up (3 months). Further longitudinal studies are necessary to evaluate the long-term effects of professional hygiene on the periodontal status of patients undergoing different types of orthodontic appliances.

## 5. Conclusions

In accordance with the null hypothesis, within the limits of the current study, it can be concluded that no evidence of difference was observed in the periodontal health of patients undergoing fixed orthodontic therapy and clear aligner therapy, when a dental hygienist provided regular check-ups and adequate oral hygiene instructions. Therefore professional oral hygiene associated with motivation and reinforcement for the adequate control of dental biofilm during the orthodontic treatment allows the patients to prevent the onset of periodontal disease and achieve good periodontal health, despite the type of orthodontic appliance used.

## Figures and Tables

**Table 1:** Characteristics of the patients included in the study for FG and CAG.

	Total	FG	CAG	*p*
*Age (years)*				
M ± SD	27.6 ± 12.6	20.6 ± 8.1	34.7 ± 12.5	**0.0001**
*Sex*				
Female	26 (65%)	11 (55%)	15 (75%)	0.180
*Number of sites*				
M ± SD	168 ± 9.1	165 ± 9.8	171 ± 7.4	**0.030**
*Gingival biotype thick*	30 (75%)	16 (80%)	14 (70%)	0.460
*Gingival biotype upper thick*	33 (82.5%)	18 (90%)	15 (75%)	0.210
*Gingival biotype lower thick*	31 (77.5%)	16 (80%)	15 (75%)	0.700

FG: fixed group; CAG: clear aligners group; M: mean; SD: standard deviation. Statistically significant differences are reported in bold.

**Table 2:** Intra-group differences after a 3 month follow-up (T1 − T0).

	FG				CAG			
	T0	T1	Δ	*p*	T0	T1	Δ	*p*
*PD, %*								
median	10.4	0	−9.2	**0.0001**	13.9	0.25	−12.6	**0.0002**
IQR	6.1, 24.2	0, 1.2	−22.5, −5.5		4.8, 31.1	0, 2.9	−25.4, −4.8	
*PI, %*								
median	30.5	14.5	−17.5	**0.040**	41.5	10.5	−24	**0.002**
IQR	5, 73	3.5, 24	−62.5, 14.5		25, 53	2, 23	−49.5, −5	
*BOP, %*								
median	77	13.5	−53.5	**0.0001**	55.5	13.5	−37.5	**0.0001**
IQR	56.5, 85	6, 28	−70.5, −37		39.5, 70	5, 17.5	−54.5, −23	
*REC, %*								
median	4.4	5.2	1.3	**0.006**	22.2	24.3	1.25	0.380
IQR	0, 14.7	2.3, 18.4	0, 3.4		7.1, 32.9	6.5, 44.5	−5.7, 7.3	

T0: baseline; T1: 3 month follow-up; Δ (delta)= T1 − T0; IQR: inter-quartile range; FG: fixed group; CAG: clear aligners group; PD: probing depth, PI: plaque index, BOP: bleeding on probing; REC: gingival recession. Statistical analysis was performed using a paired test for asymmetric distribution (Wilcoxon signed-rank test). Statistically significant differences are reported in bold.

**Table 3:** Linear regression models of difference (T1 − T0), delta (Δ), for each periodontal variable.

Outcome	Model	Beta	95% CI	*p*
Δ %PD	1	−1.8	−10.7, 7.1	0.69
	2	1.2	−1.8, 4.1	0.43
	3	2.9	−0.8, 6.7	0.12
	4	2.8	−0.9, 6.5	0.14
Δ %PI	1	−3.8	−27.6, 19.9	0.75
	2	−0.6	−12.9, 11.7	0.92
	3	−3.1	−18.5, 12.3	0.68
	4	−5.0	−20.3, 10.3	0.51
Δ %BOP	1	14.9	−0.01, 29.9	0.07
	2	−0.5	−10.6, 9.7	0.93
	3	2.0	−11.3, 15.4	0.76
	4	0.6	−12.4, 13.6	0.92
Δ %REC	1	−2.0	−7.2, 3.3	0.45
	2	−0.5	−6.2, 5.2	0.87
	3	−4.0	−10.2, 2.1	0.19
	4	−4.1	−10.1, 1.9	0.18

PD: probing depth; PI: plaque index; BOP: bleeding on probing; REC: gingival recessions, CI: confidence interval.
